# Measuring Stigma Towards People with Opioid Use Problems: Exploratory and Confirmatory Factor Analysis of the Opening Minds Provider Attitudes Towards Opioid-Use Scale (OM-PATOS)

**DOI:** 10.1007/s11469-022-00788-z

**Published:** 2022-03-03

**Authors:** Stephanie Knaak, Scott Patten, Heather Stuart

**Affiliations:** 1 Mental Health Commission of Canada, 350 Albert Street, Suite 1210, Ottawa, ON K1R 1A4 Canada; 2 University of Calgary, Calgary, AB Canada; 3 Queen’s University, Kingston, ON Canada

**Keywords:** Stigma, Psychometric testing, Stigma scale, Opioid, Healthcare provider, First responder, Canada, Exploratory factor analysis, Confirmatory factor analysis

## Abstract

Many countries are experiencing an ongoing opioid crisis characterized by high rates of opioid use problems, overdose, poisoning, and death. Stigma has been identified as a central problem for seeking and receiving quality services from health providers and first respondents. The Mental Health Commission of Canada developed a scale that could be used to measure stigma in this population, as no such scale currently exists. This paper provides the results of psychometric testing of this new scale, known as the Opening Minds Provider Attitudes Towards Opioid-Use Scale (OM-PATOS), using exploratory (EFA) and confirmatory (CFA) factor analysis. EFA findings showed a 15 item 2-factor solution, with subscales of ‘attitudes’ (6 items) and ‘behaviours/motivation to help’ (9 items). The confirmatory factor analysis provided some preliminary confirmation of the factor structure suggested by the exploratory analyses, but further research with larger samples is needed to fully confirm the factor structure. Overall, results support the use of the 15-item scale with health professionals and first responders, with factors used for descriptive value rather than as calculated subscales until further research can be completed.

The USA and Canada, along with a number of other countries, are experiencing an ongoing opioid crisis characterized by high rates of opioid use problems, overdose, poisoning, and death, a crisis which has only escalated in the context of public health responses to the COVID-19 pandemic (Degenhardt et al., 2019; Dunlop et al., 2020; Häuser et al., 2021; Salmond & Allread, 2019; Tyndall, 2020, Vojtila et al., 2019). In some parts of both the USA and Canada, the crisis has led to decreases in overall life expectancy, particularly among men, and deaths from opioids have been close to or exceeding those from COVID-19 (OECD, 2019; Orpana et al., 2019; Smith, 2020; The Economist, 2021).

Many other countries, including Australia, Sweden, Norway, Ireland, France, Finland, Ireland, Russia, Libya, and the UK have also seen marked increases in opioid use disorders, opioid-related harms, or opioid-related deaths (Degenhardt et al., 2019; Häuser et al., 2021; OECD, 2019). Worldwide, about 0.5 million deaths are attributable to drug use. More than 70% of these deaths are related to opioids (Volkov, 2021). The main drivers of these trends include the growth in the availability of prescription analgesic opioids, coupled with availability, complexity, and increasing toxicity of the illegal drug supply with the introduction of synthetic opioids such as fentanyl and carfentanil (OECD, 2019; Special Advisory Committee on the Epidemic of Opioid Overdoses, 2020).

In Canada, the emergence of fentanyl and its analogs into the non-prescription drug supply has been a central factor in the ongoing crisis, with recent data showing 87% of accidental opioid-related deaths in Canada involved fentanyl (Government of Canada, 2021). While opioid use problems and risks of overdose or poisoning occurs across all major demographic groups, the majority of opioid-related deaths in Canada tend to occur among males (75%), with the majority of deaths occurring among individuals (for both male and female) between 20 and 49 years of age (Government of Canada, 2021).

As part of Canada’s response to this crisis, the issue of stigma was highlighted as an important research need, as it was identified as a potential contributor in the avoidance of harm reduction, treatment, and other services among people who use drugs, as well as a lower quality of care and service delivery from providers (Knaak et al., 2019a, b). Indeed, research shows that people with substance use problems tend to be viewed as untrustworthy, less deserving of care, responsible for their illness, untreatable, or largely unable to recover and that such negative views are not only endorsed by the general public, but among health professionals and other direct service providers as well (Barry et al., 2014; Corrigan & Nieweglowski, 2018; Kennedy-Hendricks et al., 2016; Stuart, 2019; van Boekel et al., 2013). Also, a recent review by Stuart (2019) found considerable stigmatization towards opioid use, particularly in the context of help seeking and treatment, including methadone maintenance therapy (MMT) and other opioid agonist therapies, for example. Qualitative research with people with lived and living experience (PWLLE) of opioid use disorders supports this, with service users experiencing considerable negative interactions and treatment from health providers (Brener et al., 2010; Earnshaw et al., 2013; Woo et al., 2017).

Attending to the problem of stigmatization towards people with opioid use problems is thus an important priority, both at the structural level of policy, access, and availability of services (Livingston, 2020) and also at the interpersonal and individual level of service provision—such as improving the quality of client-provider interactions, levels of client satisfaction, experiences of help seeking, and retention in services or care (Knaak et al., 2019a, b; Stuart, 2019; Volkow, 2020).

To assist researchers and others in better understanding and addressing issues related to the stigmatization of people who use opioids in the context of health and direct service provision, researchers working with the Mental Health Commission of Canada developed a scale that could be used to measure stigma in this population, as no such scale currently exists. The rationale for the design of such a scale is that it could be used to gauge attitudes and behaviours of direct service providers and help assess the impact of stigma reduction initiatives, interventions, or programs. This paper provides the results of psychometric testing of this new scale, known as the Opening Minds Provider Attitudes Towards Opioid-Use Scale (OM-PATOS).

## Background on Scale Development

The OM-PATOS was developed as part of a larger project sponsored by the Mental Health Commission of Canada which sought to (a) understand problem of stigmatization in the context of Canada’s opioid crisis with specific attention to stigma among direct service providers towards people with opioid use disorders and people at risk of opioid-related overdoses and poisoning and (b) identify and evaluate promising approaches and strategies for stigma reduction in direct service environments (Knaak et al., 2019a, b, 2020, 2021).

Scale development activities took place in 2018–2019 and have been described in detail in another publication (Knaak & Stuart, 2022). They are summarized as follows. First, eight focus groups and 15 key informant interviews were conducted with various direct service provider groups across Canada—including paramedics, fire services, police services, health providers, social service and outreach workers, policy and program staff, and people with lived and living experience of substance use. Results from the focus groups and interviews were then used to generate the main domains for the OM-PATOS—‘attitudes’ and ‘behaviours/orientation towards caring’. Using these domains as a foundation, existing measures were reviewed to generate an initial item pool, with specific attention to measures that captured aspects of substance use stigma as well as measures that captured attitudes and orientation of helping professionals towards people with substance use problems or mental illnesses.

Following the generation of an item pool, three expert consultation group meetings were conducted with service providers, PWLLE, and research experts for review of proposed items and response anchors. Each expert consultation meeting comprised between five and eight participants. Expert consultations were followed by a series of four cognitive interviews with the refined scale to ensure that interpretation of the items was as designed. One cognitive interview was completed with a person with lived experience of opioid use. The other three cognitive interviews were with direct service providers—a police officer, a health provider, and an addictions counsellor.

The results of the expert consultations suggested a five-point Likert scale ranging from strongly disagree (1) to strongly agree (5) as the preferred response anchor, with statements worded to the negative such that lower scores (i.e. disagreement with the statement) indicated a lower level of stigma. The draft scale was then pilot tested with a convenience sample of 56 service providers, which led to further refinements, resulting in a 24-item scale with a Cronbach alpha of 0.87 (as based on the pilot test sample). A 4-week test–retest analysis of the pilot sample (*n* = 37) revealed an interclass correlation coefficient (ICC) of 0.79, suggesting acceptable scale stability. Additional details on the development of the OM-PATOS are described in Knaak and Stuart (2022).

In early 2020, a preliminary exploratory factor analysis (EFA) was completed on the 24-item scale with a sample of practicing and student participants in the health and allied health fields (e.g. nursing, social work, pharmacy, addiction counselling, administration/management) who had enrolled in an online anti-stigma training program (*n* = 460), the results of which suggested a possible single factor solution with 19 items, explaining 56.9% of the variance (unpublished data). While the use of the 19-item scale showed strong reliability (Cronbach alpha = 0.96) and good responsiveness to change when used in real-world settings (e.g. Knaak et al., 2021), there was a desire to revisit the psychometric properties of the OM-PATOS and undertake additional testing. The reasons were three-fold. First, the sample pool from the initial exploratory factor analysis was not large enough to be able to undertake both exploratory and confirmatory factor analyses, which is the recommended best practice for verifying scale factorial validity (Souza et al., 2017). Second, the sample pool from the initial exploratory factor analysis had a high proportion of students and people working in management and administration roles, which was not ideal as the scale itself was developed primarily for practicing healthcare professionals and first responders. And third, a single factor solution would fail to distinguish the theoretical dimension of attitudes from that of behaviours/orientations towards caring, which emerged as important within the scale development process (see Knaak & Stuart, 2022) and which is also discussed as an important consideration for stigma, as behaviours and attitudes, while connected, are also distinct (Stuart, 2016).

The remainder of this paper describes the psychometric testing of the 24-item scale using an exploratory factor analysis, followed by a confirmatory factor analysis on a sample of health professionals and first responders.

## Methods

### Sample

The testing sample was generated using baseline data from four different anti stigma programs that employed the 24-item OM-PATOS as an evaluation tool. Three of these programs were delivered in person, and one was web-based. The programs were being evaluated for their effectiveness at reducing stigma towards people with opioid use problems in the context of health and social care. Further details about each of the four interventions can be found in Kharpal et al. (2021). For the evaluation studies, the 24-item OM-PATOS survey was administered to each audience prior to the anti-stigma intervention being received, with a post-test administered afterwards. Baseline demographic information was also collected, including gender, age, and profession (see Table 1). Participants represented diverse types of direct service providers. For the current analysis, we included all participants who identified working in a health profession (e.g. physician, nurse), allied health (e.g. social work, pharmacy, addiction counsellor), or first responder role (e.g. paramedic, police, fire fighter). Testing of psychometric properties was completed for respondents who fully completed the OM-PATOS baseline survey (*n* = 822). To undertake the analyses, the sample was randomly divided into two separate files, with approximately half of the cases (*n* = 432) used for the EFA and half of the cases (*n* = 390) used for the subsequent CFA.

**Table 1:** Sample characteristics

	Full sample *n* (%)*	EFA *n* (%)*	CFA *n* (%)*
Gender			
Male	133 (17.0%)	77 (18.6%)	56 (15.1%)
Female	648 (82.8%)	335 (81.1%)	313 (84.6%)
Non-binary	2 (0.3%)	1 (0.3%)	1 (0.3%)
Missing	39	19	19
Age	*M* = 36.9	*M* = 36.9	*M* = 36.9
25 and under	152 (19.8%)	75 (18.4%)	178 (21.3%)
26–35	249 (32.4%)	134 (33.3%)	115 (31.4%)
36–45	164 (21.4%)	95 (23.6%)	69 (18.9%)
46–55	133 (17.3%)	59 (14.7%)	74 (20.2%)
56–65	61 (7.9%)	34 (8.5%)	27(7.4%)
Over 65	9 (1.2%)	6(1.5%)	3 (0.8%)
Missing	54	30	24
Occupation			
Nurse	349 (42.5%)	177 (41.0%)	172 (44.1%)
Physician	16 (1.9%)	8 (1.9%)	8 (2.1%)
Allied health (e.g. pharmacist, social work, rehabilitation therapist, addiction counsellor)	376 (45.7%)	202 (46.8%)	174 (28.8%)
First responder (fire, paramedic, law enforcement)	81 (9.8%)	45 (10.4%)	36 (9.3%)

^*^Valid percent reported

### Exploratory Factor Analysis (EFA)

The EFA was conducted using principal axis factoring, as the aim was to help determine the underlying theoretical structure of the scale (Henson & Roberts, 2006). Factors were extracted using Horn’s parallel analysis (Hayton et al., 2004), eigenvalue-one procedure, the requirement that each factor must contain three or more items to be retained, and seasoned reflection (Henson & Roberts, 2006). Items with a loading of greater than 0.45 were retained for a specific factor (Boateng et al., 2018). Items that loaded onto more than one factor were removed (Boateng et al., 2018). An oblique (promax) rotation method was selected as the preferred rotation structure recommended for social science research, as it is assumed that the underlying factors are correlated (Costello & Osborne, 2005; Schmitt, 2011). Internal consistency was evaluated using the Cronbach’s alpha coefficient and the Omega statistic (Nunnally & Bernstein, 1994; Trizano-Hermosilla & Alvarado, 2016).

Prior to undertaking the EFA, Bartlett’s Test of Sphericity and Kaiser–Meyer–Olkin (KMO) measure of sampling adequacy were calculated to verify the appropriateness of using factor models (Hadi et al., 2016).

### Confirmatory Factor Analysis (CFA)

The confirmatory factor analysis was conducted using structural equation modelling (SEM) in Stata v17 (College Station, Texas). This analysis sought to confirm the factor structure suggested by the exploratory factor analysis: a six item ‘attitudes’ factor (items 3, 5, 9, 10, 12, and 21) and a nine item ‘behaviours/motivation to help’ factor (items 4, 7, 11, 14, 17, 18, 20, 22, and 23). The analysis was conducted using 390 observations that were not used in the exploratory factor analysis, as described above. These two factors were allowed to correlate with each other.

During data analysis, the questionnaire items were found not to be normally distributed. Negative response items (‘strongly disagree’) tended to be those most frequently endorsed. Unfortunately, the sample size was insufficient to obtain reliable results from asymptotically distribution-free methods, which require sample sizes > 1000 (Schermelleh-Engel et al., 2008). Therefore, following recommendations from Schermelleh-Engel et al. (2008), maximum likelihood estimation was used, but with robust standard errors to at least partially address the violated distributional assumption. Goodness of fit was assessed using the fit statistics provided by Stata (College Station, TX n.d.) for this type of estimation: (1) standardized root mean squared residual (SRMR), which should be less than 0.08, and (2) the coefficient of determination, which should be close to 1.

## Results

### Exploratory Factor Analysis

The Kaiser–Meyer–Olkin (KMO) measure of sampling adequacy was 0.95, which is considered excellent (Hadi et al., 2016). The Bartlett’s Test of Sphericity was statistically significant (*χ*2 = 6562.40, *p* < 0.001), supporting the factorability of the correlation matrix. No indications of collinearity were observed, with all correlations being below 0.90. The initial EFA of the 24 items showed four factors with eigenvalues above 1.0; however, only two items were loaded onto the third factor (13, 19), and only one item loaded onto the fourth factor (item 19), failing to meet the minimum requirement of three or more items per factor. Also, the results of the parallel analysis suggested that a two-factor solution may be preferred. These items were removed, and the EFA was repeated. In the repeated EFA, four items (1, 6, 8, 24) did not load onto any factor. Each item was tested for deletion individually. All items were subsequently removed, and the EFA was rerun. In the repeated analysis, item 2 did not load onto any factor. This item was removed, and the EFA was repeated.

Table 2 shows the results of the EFA upon removal of the identified items. As shown, the remaining 15 items were loaded onto two factors—labelled ‘attitudes’ (items 3, 5, 9, 10, 12, 21) and ‘behaviours/motivation to help’ (items 4, 7, 11, 14, 17, 18, 20, 22, 23). This solution explained 60.62% of the variance. Both the Cronbach alpha and the Omega statistic were 0.87 for the ‘attitudes’ factor and 0.94 for the ‘behaviours/motivation to help’ factor, indicating very good reliability. Table 3 provides the sample means and item-total correlations for the 15 scale items.

**Table 2:** Exploratory factor analysis

	Pattern matrix		Structure matrix		
	Behaviours/motivation to help	Attitudes	Behaviours/motivation to help	Attitudes	*h*^2*^
Q3. People with opioid use problems are to blame for their problems	.341	**.487**	.725	.756	.615
Q4. I tend to use negative terms to talk about people with opioid use problems	**.790**	.007	.795	.631	.633
Q5. People with opioid use problems cost the system too much money	.223	.**484**	.606	.660	.455
Q7. I tend to act more negatively towards people with opioid use problems than other people I help	.813	− .065	.762	.577	.582
Q9. People with opioid use problems cannot be trusted	− .028	.**776**	.585	.754	.569
Q10. People with opioid use problems who take drug therapies like methadone are replacing one addiction with another	− .109	.**818**	.537	.732	.540
Q11. I tend to be less patient towards people with opioid use problems than other people I help	.**642**	.106	.726	.613	.531
Q12. People with opioid use problems only care about getting their next dose of drugs	.100	.754	.695	.832	.697
Q14. When people with opioid use problems ask for help with something, I have a hard time believing they are sincere	.**504**	.348	.780	.747	.653
Q17. I tend to negatively judge people with opioid use problems	.**877**	.005	.882	.699	.778
Q18. People with opioid use problems who relapse while trying to recover are not trying hard enough to get better	.**578**	.219	.7513	.675	.582
Q20. I tend to speak down to people with opioid use problems	**.739**	.078	.801	.662	.644
Q21. Most people with opioid use problems engage in crime to support their addiction	.102	.**624**	.595	.705	.501
Q22. If a co-worker says something negative about people with opioid use problems, I would be more likely to speak negatively when discussing them myself	.**786**	− .050	.747	.571	.559
Q23. I tend to think poorly about people with opioid use problems	.**693**	.211	.860	.759	.757

^*^h^*2*^ = communality coefficient. Extraction method: Principal axis factoring. Rotation method: Promax with Kaiser normalization. Total variance explained 60.62% (56.56% factor 1, 4.07% factor 2); Eigenvalues for the two factors were 8.86 (factor 1) and 1.02 (factor 2)

**Table 3:** Scale item descriptives

	Mean	SD	Item-total correlation
Q3. People with opioid use problems are to blame for their problems	1.88	1.01	.760
Q4. I tend to use negative terms to talk about people with opioid use problems	1.84	.974	.745
Q5. People with opioid use problems cost the system too much money	2.30	1.110	.648
Q7. I tend to act more negatively towards people with opioid use problems than other people I help	1.91	.992	.701
Q9. People with opioid use problems cannot be trusted	2.29	1.011	.667
Q10. People with opioid use problems who take drug therapies like methadone are replacing one addiction with another	2.16	1.108	.624
Q11. I tend to be less patient towards people with opioid use problems than other people I help	1.93	.974	.697
Q12. People with opioid use problems only care about getting their next dose of drugs	2.06	.971	.767
Q14. When people with opioid use problems ask for help with something, I have a hard time believing they are sincere	1.94	.954	.785
Q17. I tend to negatively judge people with opioid use problems	1.91	.964	.822
Q18. People with opioid use problems who relapse while trying to recover are not trying hard enough to get better	1.67	.859	.737
Q20. I tend to speak down to people with opioid use problems	1.73	.910	.759
Q21. Most people with opioid use problems engage in crime to support their addiction	2.32	1.032	.656
Q22. If a co-worker says something negative about people with opioid use problems, I would be more likely to speak negatively when discussing them myself	1.99	.989	.688
Q23. I tend to think poorly about people with opioid use problems	1.87	.898	.840

### Confirmatory Factor Analysis (CFA)

The extent of missing data in the validation sample was small. Eighteen respondents (2.9%) who did not report their occupation were excluded from the analysis. Two participants (0.5%) were excluded from the CFA due to missing data on questionnaire items. The CFA was conducted using data from 388 participants having complete data.

The fit statistics are summarized in Table 4. The confirmatory factor analysis provided some preliminary confirmation of the factor structure suggested by the exploratory analyses. The SRMR was 0.036, and the coefficient of determination was 0.981.

**Table 4:** Confirmatory factor analysis fit statistics for 3 study populations

	Health professionals and first responders (*n* = 388*)
Likelihood ratio *χ*2 test (vs. saturated model)	*χ*2 = 320.175, *df* = 89, *p* < 0.001
RMSEA	0.082
CFI	0.945
TLI	0.935
SRMR	0.036

^*^2 observations were excluded due to missing data scale

## Discussion

This study examined the factor structure of the OM-PATOS scale with health providers and first responders using both exploratory and confirmatory techniques. Psychometric analyses focussed on specifically on these population groups as the scale was designed specifically for use with people who work in helping professions who may respond to or care for people with opioid use problems or who may be at risk of experiencing an overdose or poisoning (e.g. paramedics, fire services, police services, health providers, social workers, pharmacists, counsellors).

Findings from the exploratory factor analysis led to the extraction of a 2-factor solution of the original 24 items, suggesting a 15-item version with subscales of ‘attitudes’ (6 items) and ‘behaviours/motivation to help’ (9 items). Internal consistency was very good for both factors. The confirmatory factor analysis provided some preliminary confirmation of the two-factor model suggested by the exploratory factor analysis in health professionals and first responders. However, it was only possible to perform a preliminary confirmation of the factor structure as the sample size was too small to support asymptotic distribution-free fitting of the hypothesized two factor model.

As the scale was conceptually designed to assess negative attitudes and behaviours/orientation towards care, these preliminary findings are encouraging. The scale’s items were meaningful to participants, had good internal consistency, and were sensitive change. Also, encouraging results were found with maximum likelihood estimation fit using robust standard errors. However, as the item responses are ordinal, distribution-free methods with larger samples will be needed to further explore and confirm the factor structure. Until such further research can be undertaken, the two conceptual dimensions of 15-item OM-PATOS should be used only for descriptive value, and not to produce subscale scores. Also, while results support the use of the OM-PATOS with health and social care providers and first responders who provide direct services to clients, we do not recommend its use in other population groups, as further research is first needed with larger and more diverse samples to understand measurement properties of the scale and its validity in other settings and populations.

## Conclusion

The OM-PATOS was developed to measure stigma towards people with opioid use problems in the health provider and first responder populations. Previous versions of the scale have been used to help assess the impact of substance and opioid use-related anti-stigma interventions targeting direct service providers and have shown good reliability and responsiveness to change in these contexts. The results of this study suggest a 15-item scale to assess stigma towards people with opioid use problems in practicing health provider and first responder populations. While the study provides preliminary support of a hypothesized two factor structure for ‘attitudes’ and ‘behaviours/motivation to help’, further research with larger samples is needed before the factor structure can be confirmed.

## References

[CR1] Barry CL, McGinty EE, Pescosolido BA, Goldman HH (2014). Stigma, discrimination, treatment effectiveness, and policy: Public views about drug addiction and mental illness. Psychiatric Services.

[CR2] Boateng GO, Neilands TB, Frongillo EA, Melgar-Quiñonez HR, Young SL (2018). Best practices for developing and validating scales for health, social, and behavioral research: A primer. Frontiers in Public Health.

[CR3] Brener L, von Hippel W, von Hippel C, Resnick I, Treloar C (2010). Perceptions of discriminatory treatment by staff as predictors of drug treatment completion: Utility of a mixed methods approach. Drug and Alcohol Review.

[CR4] Corrigan PW, Nieweglowski K (2018). Stigma and the public health agenda for the opioid crisis in America. International Journal of Drug Policy.

[CR5] Costello AB, Osborne JW (2005). Best practices in exploratory factor analysis: Four recommendations for getting the most from your analysis. Practical Assessment, Research, and Evaluation.

[CR6] Degenhardt L, Grebely J, Stone J, Hickman M, Vickerman P, Marshall B, Bruneau J, Altice FL, Henderson G, Rahimi-Movaghar A, Larney S (2019). Global patterns of opioid use and dependence: Harms to populations, interventions, and future action. Lancet.

[CR7] Dunlop A, Lokuge B, Masters D, Sequeira M, Saul P, Dunlop G, Ryan J, Hall M, Ezard N, Haber P, Lintzeris N, Maher L (2020). Challenges in maintaining treatment services for people who use drugs during the COVID-19 pandemic. Harm Reduction Journal.

[CR8] Earnshaw V, Smith L, Copenhaver M (2013). Drug addiction stigma in the context of methadone maintenance therapy: An investigation into understudied sources of stigma. International Journal of Mental Health and Addiction.

[CR9] Government of Canada. (2021). Opioid- and stimulant-related harms in Canada [website]. https://health-infobase.canada.ca/substance-related-harms/opioids-stimulants/. Accessed January 3, 2022.

[CR10] Hadi, N., Abdullah, N., & Sentosa, I. (2016). An easy approach to exploratory factor analysis: Marketing perspective. *Journal of Educational and Social Research, 6*(1), 215. Retrieved September 9, 2021 from https://www.mcser.org/journal/index.php/jesr/article/view/8799.

[CR11] Häuser W, Buchser E, Finn DP, Dom G, Fors E, Heiskanen T, Jarlbaek L, Knaggs RD, Kosek E, Krcevski-Škvarč N, Pakkonen K, Perrot S, Trouvin AP, Morlion B (2021). Is Europe also facing an opioid crisis? A survey of European Pain Federation chapters. European Journal of Pain.

[CR12] Hayton JC, Allen DG, Scarpello V (2004). Factor retention decisions in exploratory factor analysis: A tutorial on parallel analysis. Organizational Research Methods.

[CR13] Henson RK, Roberts JK (2006). Use of exploratory factor analysis in published research. Educational and Psychological Measurement.

[CR14] Kennedy-Hendricks A, Busch SH, McGinty EE, Bachhuber MA, Niederdeppe J, Gollust SE, Webster DW, Fiellin DA, Barry CL (2016). Primary care physicians’ perspectives on the prescription opioid epidemic. Drug and Alcohol Dependence.

[CR15] Kharpal, K., Knaak, S., Benes, K., & Bartram, M. (2021). *Reducing opioid and substance use-related stigma in health-care and other direct service delivery contexts*. Ottawa, Canada: Mental Health Commission of Canada. https://mentalhealthcommission.ca/resource/reducing-opioid-and-substance-use-related-stigma-in-health-care-and-other-direct-service-delivery-contexts-evaluation-results-from-four-programs/.

[CR16] Knaak, S., Christie, R., Mercer, S., & Stuart, H. (2019a). *Stigma and the opioid crisis -- Final report*. Mental Health Commission of Canada.

[CR17] Knaak, S., Christie, R., Mercer, S., & Stuart, H. (2019b) Harm reduction, stigma and recovery: Tensions on the front-lines of Canada’s opioid crisis. *JMHAN*, *3*(1). 10.22374/jmhan.v3i1.37.

[CR18] Knaak S, Patten S, Sandrelli M (2020). How a shared humanity model can improve provider wellbeing and client care: An evaluation of Fraser Health’s Trauma and Resiliency Informed Practice (TRIP) training program. Healthcare Management Forum.

[CR19] Knaak S, & Stuart H. (2022). Measuring opioid related stigma. In Dobson, K. S., & Stuart, H. (eds.), *The Stigma of Mental Illness* (pp. 71-80). Oxford University Press.

[CR20] Knaak, S., Besharah, J., Billet, M., Kharpal, K., & Patten, S. (2022). Measuring impacts of curricular content and personal story on substance use stigma. *Journal of Nursing Education*, in press. 10.3928/01484834-20220303-08 35522772

[CR21] Livingston JD (2020). Structural stigma in health-care contexts for people with mental health and substance use issues: A literature review.

[CR22] OECD. (2019). Addressing problematic opioid use in OECD countries OECD Health Policy Studies OECD Publishing 10.1787/a18286f0-en.

[CR23] Orpana HM, Lang JJ, George D, Halverson J (2019). At-a-glance - the impact of poisoning-related mortality on life expectancy at birth in Canada, 2000 to 2016. Health Promotion and Chronic Disease Prevention in Canada: Research, Policy and Practice.

[CR24] Salmond S, Allread V (2019). A population health approach to America’s opioid epidemic. Orthopaedic Nursing.

[CR25] Schermelleh-Engel K, Moosbrugger H, Müller H (2008). Evaluating the fit of structural equation models: Tests of significance and descriptive goodness-of-fit measures. Methods of Psychological Research Online.

[CR26] Schmitt TA (2011). Current methodological considerations in exploratory and confirmatory factor analysis. Journal of Psychoeducational Assessment.

[CR27] Souza, A. C., Alexandre, N., & Guirardello, E. B. (2017). Psychometric properties in instruments evaluation of reliability and validity. Propriedades psicométricas na avaliação de instrumentos: avaliação da confiabilidade e da validade. *Applications of Epidemiology. Epidemiologia e servicos de saude : revista do Sistema Unico de Saude do Brasil, 26*(3), 649–659. 10.5123/S1679-49742017000300022.10.5123/S1679-49742017000300022 28977189

[CR28] Smith, A. (2020). Alberta's opioid deaths soar during COVID-19 pandemic in deadliest year ever. *Calgary Herald*. https://calgaryherald.com/news/politics/opioid-overdose-deaths-soar-in-alberta-marking-the-deadliest-year-ever-recorded.

[CR29] Special Advisory Committee on the Epidemic of Opioid Overdoses. (2020). Opioid-related harms in Canada*.* Ottawa: Public Health Agency of Canada. https://health-infobase.canada.ca/substance-related-harms/opioids.

[CR30] Stata. (n.d.). Version 17. *Goodness of Fit Statistics*, College Station, Texas.

[CR31] Stuart H (2016). Reducing the stigma of mental illness. Global Mental Health (Cambridge, England).

[CR32] Stuart H (2019). Managing the stigma of opioid use. Healthcare Management Forum.

[CR33] The Economist. (2021). The other epidemic. *The Economist*. https://www.economist.com/united-states/2021/05/15/last-year-more-people-in-san-francisco-died-of-overdoses-than-of-covid-19.

[CR34] Trizano-Hermosilla T, Alvarado JM (2016). Best alternatives to Cronbach’s alpha reliability in realistic conditions: Congeneric and asymmetrical measurements. Frontiers in Psychology.

[CR35] Tyndall M (2020). Safer opioid distribution in response to the COVID-19 pandemic. International Journal on Drug Policy.

[CR36] van Boekel LC, Brouwers EP, van Weeghel J, Garretsen HF (2013). Stigma among health professionals towards patients with substance use disorders and its consequences for healthcare delivery: Systematic review. Drug and Alcohol Dependence.

[CR37] Vojtila, L., Pang, M., Goldman, B., Kurdyak, P, & Fischer, B. (2019). Non-medical opioid use, harms and interventions in Canada – A 10-year update on an unprecedented and unabating substance use-related public health crisis. *Drugs: Education, Prevention, Policy*, *27*(2), 118–22.

[CR38] Volkov, S. (2021). Opioid overdose (web page). World Health Organization. https://www.who.int/news-room/fact-sheets/detail/opioid-overdose.

[CR39] Volkow ND (2020). Stigma and the toll of addiction. New England Journal of Medicine.

[CR40] Woo, J., Bhalerao, A., Bawor, M., Bhatt, M., Dennis, B., Mouravska, N., Zielinski L., & Samaan, Z. (2017). “Don’t judge a book its cover”: A qualitative study of methadone patients’ experiences of stigma, *Substance Abuse: Research and Treatment*, *11*. 1178221816685087. 10.1177/1178221816685087. 10.1177/1178221816685087 PMC5398333 28469424

